# Letter from Philadelphia

**Published:** 1862-03

**Authors:** 


### Correspondence.

Messrs. Editors :—I am very sorry that anything I have written should have been made the occasion or a pretext for the Editorial comment on the Faculty of the "Pennsylvania College of Dental Surgery," which appeared in your January number.

These Editorial remarks were as entirely unwarranted as uninvited; and as will be plainly seen, my language had to be disjointed and perverted, to give even an apparent basis for this display of ill feeling, which amounts to positive injustice, and I must say, insult; and such will be the verdict, I am sure, of every unprejudiced mind.

*Correction.*—I wish to make a correction in my article published in your January number. In that article, alluding to the productions of a certain writer, I say, "for all his papers abound in just such jargon." Now, I wish to qualify this by the addition of the word "late" before papers, as it is quite possible the gentleman may have written much that I have never seen.

*The Commencement* exercises of the "Philadelphia College of Dental Surgery" took place last evening, February 28th, at the Musical Fund Hall, in presence of a very large audience.

The graduates number nineteen out of a class of forty-four. The Valedictory,—a very appropriate though rather lengthy address—was delivered by Prof. J. L. Suesserott.

The Demonstraters' report shows six hundred and twenty patients in the operative department, for whom thirty-five hundred and ninety-seven operations were performed, and in the mechanical department twelve hundred and ten teeth mounted during the session.

Did you ever think of the annoyance and loss of time, caused by some in our profession, by compelling their patients to call several times, where one, or two visits at most, should have been sufficient? In looking for a motive for this too prevalent practice in cities, two things occur to me. First, that the operator wishes to impress upon the minds of his patients an undue sense of his importance and the service he is rendering them, and with which the charges usually agree. Second, to keep his reception-room occupied, and thus give the appearance of a large practice.

Some years ago I frequently called upon a very gentlemanly dentist in a neighboring city when passing through it, and always found a number of persons in his waiting-room, and therefore took it for granted that he was in the enjoyment of a large practice, and still he was always finanically embarrassed. I inquired of a friend in the same city and neighborhood—a dentist—the reason why Dr. ——— could not get along, as he evidently had a large practice; his reply was,—"That man is a false pretense, he has but a limited practice, his bad work, and more especially his practice of making his patients call half-a-dozen times to have done what should and could be done at one sitting, keeps his parlor full all the time, but his receipts are not in proportion to appearances."

I know two gentlemen in our profession in this city, one of whom practices on this principle, and go when you will during the day into his parlor, you will always find a person or persons in waiting. With the other, his chair and parlor are often vacant, yet the latter, I have reason to believe, has by far the largest revenue, while both are good operators. Appearances, therefore, are deceptive.

A serious objection to this draft upon the time and patience of patients, aside from its impropriety, is the erroneous idea they very naturally form of the profession, and the association in their minds of repeated and painful visits, and which is almost enough, and in many instances quite sufficient, to deter them from having anything further done by the dentist, thus preventing the accomplishment of the very object sought to be gained by those who practice these little deceptions,—these tricks of trade—and also damaging to the character of the profession. Yours, O. U. C.

*Philadelphia, March* 1, 1862.

